# Supplementary material for: Content-rich biological network constructed by mining PubMed abstracts
Source: BMC Bioinformatics. 2004 Oct 8;5:147. doi: 10.1186/1471-2105-5-147 (PMC528731; doi:10.1186/1471-2105-5-147)
Supplement: Additional File 2 — The original results of the above study (non-essential files are deleted to keep the file size under the limit set by BMC bioinformatics). [file 1471-2105-5-147-S2.bz2 › chilibotAdditionalFile2/dip05/402ID10026196E10012/html/IL11_IL6ST.html]

 


 **IL11** and **IL6ST** 
  
Found 219 abstracts in PubMed, retrieved 05.  
 

 What does Google say? 
 PDF only 
| .edu only 

---

**Interactive relationship** (e.g. stimulation, inhibition, etc)

**Neutral relationship**- IL 11  [ **IL11** ]  forms a high affinity complex, thereby inducing gp130  [ **IL6ST** ]  dependent signaling.  Ref: 12865319 Endocrinology, 2003
- IL 11  [ **IL11** ]  is a member of the gp130  [ **IL6ST** ]  family of cytokines, which signal via assembly of multisubunit receptor complexes containing at least one molecule of the transmembrane signaling receptor gp130  [ **IL6ST** ] .  Ref: 12865319 Endocrinology, 2003

**Non-interactive relationship** (e.g. studied together, co-existance, homology, etc.)

- OnM induced desensitization also prevents responses to other gp130  [ **IL6ST** ]  signaling cytokines e.g. leukemia inhibitory factor and interleukin 11  [ **IL11** ] .  Ref: 12724316 J Biol Chem< MedlineTA>J Biol Chem, 2003
- ELISA was used to determine the serum levels of soluble interleukin 2 receptors, interleukin 1  [ **IL11** ] , interleukin 6, and 2 soluble interleukin 6 receptors sIL 6R and soluble gp130  [ **IL6ST** ]  in 18 patients with cluster headache 6 women and 12 men during the cluster period and in 17 healthy controls who were headache free 3 women and 14 men.  Ref: 12864761 Headache, 2003
- Previous studies have identified three distinct receptor binding sites, I, II, and III, crucial for the binding of murine IL 11  [ **IL11** ]  mIL 11 to both the IL 11R and gp130  [ **IL6ST** ] .  Ref: 12865319 Endocrinology, 2003
